# The impact of correctional officer gender on prison suicide

**DOI:** 10.1186/s40352-023-00214-z

**Published:** 2023-02-21

**Authors:** TaLisa J. Carter, Tanya N. Whittle

**Affiliations:** 1 Department of Justice, Law, and Criminology, American University, Massachusetts Avenue, NW Washington, DC 20016 USA; 2 Department of Sociology and Criminal Justice, Delaware State University, Dover, USA

**Keywords:** Correctional Officer, Gender, Suicide, Deprivation, Importation

## Abstract

Correctional officers are critical members of the prison community. However, scholarship rarely considers how correctional officers contribute to prison outcomes instead largely focusing on importation (individual) and deprivation (organizational) factors related to the incarcerated population. This is also true regarding how scholars and practitioners approach suicide committed by incarcerated people, one of the leading causes of death in US carceral institutions. Using quantitative data from confinement facilities across the United States, this study answers the research question: What is the relationship between prison suicide rates and correctional officer gender? Results show that deprivation factors (variables related to the carceral environment) influence prison suicide. Additionally, gender diversity among correctional officers reduces the rate of prison suicide. Implications for future research and practice and limitations of the study are also discussed.

In recent years, one of the most challenging issues in corrections is the uptick in prison suicide rates (Carson, 2021a, 2021b; Noonan, 2016). Suicide is the leading cause of death in jails; for state prisoners, suicide is the leading cause of unnatural death (Carson, 2021a, 2021b; Noonan & Ginder, 2014). In response, professional correctional organizations adopt policies that consider officers a focal point for suicide prevention programming (Danto, 1997; Hayes, 1995). Likewise, researchers who explore prison suicide recommend advancing the training and knowledge of correctional staff to improve responses to suicidal ideation and/or behaviors (Forrester & Slade, 2014; Slade & Forrester, 2015). In short, practitioners and researchers overwhelmingly agree that correctional officers are key in suicide prevention. This makes sense, as correctional officers are the first responders to detainees who demonstrate suicidal ideation or behaviors. However, viewing correctional officers as a singular body working to prevent suicide in a uniform way fails to acknowledge their complex and diverse nature. This includes failing to acknowledge the impact diverse officer demographics such as gender may have on prison suicide. This study focuses on the impact of the gender composition of correctional officers on prison suicide.

It is expected that the gender of correctional officers will impact corrections outcomes because women are likely to have humanistic orientations (i.e., treat incarcerated people humanely) in comparison to their male counterparts (Gordon, 2006). This expectation was built into the structure of corrections since women began working as officers in the United States in 1882 (Crank, 2015). At that time, female officers focused on instructing incarcerated women on biblical study and domestic tasks (Crank, 2015). In the 142 years since, the numbers of women in corrections expanded to 28.2% of the workforce and their assignments have diversified as well. Female officers now routinely interact with male detainees under a variety of challenging conditions including mental health wings, administrative segregation, and solitary confinement. Despite these changes, the role of women in corrections is still grounded in humanistic orientations (Martin & Jurik, 2007). For example, Britton’s (2006) work finds that assignments of correctional officers can vary by gender. Supervisors who doubt the ability of female officers may assign simple tasks or those that require minimum physicality (Britton, 2006). In other words, corrections continue to consider women’s ability in the profession to be nurturing and humanistic. Therefore, it is expected that the presence of female correctional officers will reduce suicide rates because their interactions are more likely to be humane and rehabilitative than harsh and retributive, yet research to date have not explored this area.

This study directly addresses this gap in the literature by exploring the relationship between correctional officer gender and prison suicide rates. Previous literature focuses on how correctional officer training (Slade & Forrester, 2015), individual (i.e., incarcerated people), and environmental factors contribute to the likelihood of prison suicide (Daniel & Fleming, 2006; Dye, 2010). Yet, studies fail to acknowledge the potential importance of officer gender on prison suicide rates. This is surprising as female correctional officers are more likely to rely on interpersonal communication and other humanistic skills when interacting with incarcerated people (Gordon, 2006). Therefore, it is expected that the presence of female correctional officers will translate to a less punitive approach in interacting with incarcerated people. First, this paper briefly examines factors applied to prison suicide in previous literature relying on two theoretical frameworks: importation and deprivation. It then reviews the gendered nature of correctional employment, the effects of gender on the prison environment, and how these dynamics connect to prison suicide. Using quantitative data from confinement facilities across the United States, this study then answers the research question: What is the relationship between prison suicide rates and correctional officer gender? The paper concludes with a discussion of the importance of gender diversity among correctional officers and translational suggestions for future research and practice.^1^

## Literature review

### Understanding suicide among incarcerated people

From 2001–2019, 4525 deaths by suicide occured in Bureau of Prison state and federal prison facilities (Carson, 2021a, 2021b), with the annual figure holding relatively steady in 2018 (311) and 2019 (312). In terms of local jails, 49 per 100,000 deaths are the result of suicide; it is the leading cause of fatalities in custody. Considering these staggering rates, scholarship that examines suicide among incarcerated populations is vital. The theoretical frameworks that are most consistently relied upon to understand these phenomena are importation and deprivation. This section reviews the literature through the lenses of these frameworks.

The importation model takes the perspective that prison culture reflects characteristics that detainees bring into facilities (Carroll, 1974; Irwin & Cressey, 1962; Jacobs, 1974; Wacquant, 2001). According to this perspective, individual level factors shape prison culture. Among incarcerated people, males are more likely to commit suicide in prison than females (Lester & Danto, 1993; Salive et al., 1989; White et al., 2002). Younger incarcerated people are at a greater risk to commit suicide than their older counterparts (Lester & Danto, 1993; Alison Liebling, 1999). Race of incarcerated persons is also significantly related to prison suicide; White people who are confined are more likely to commit suicide than their minority counterparts (Anno, 1985; Anson & Cole, 1984; Daniel & Fleming, 2006; Danto, 1973; Haycock, 1989; Kovasznay et al., 2004; Mumola, 2005; Rodgers, 1995; Salive et al., 1989; Way et al., 2005; White et al., 2002). Prison suicide is also correlated with the psychological condition of detainees (Anno, 1985; Bland et al., 1998; Dooley, 1990; Kovasznay et al., 2004; Way et al., 2005; White et al., 2002). Incarcerated individuals who were receiving mental health services comprised 84 percent of the suicide cases that happened in New York between 1993–2000 (Kovasznay et al., 2004). Way et al. (2005) also examined data from carceral facilities in New York and found that 41% of individuals who committed suicide received mental health services three days before the event. Scholars use individual traits to develop a profile describing those that are most likely to commit suicide during incarceration. Specifically, in a systematic review of suicides within one state, Daniel and Fleming (2006) find that prisoners who are young, single, White, and male, who struggle with mental health/substance abuse problems are more likely to commit suicide. In short, scholarship justifies including individual-level (detainee) characteristics when trying to determine what factors influence prison suicide rates.

In contrast to importation, the deprivation framework focuses on how organizational factors influence corrections outcomes. The deprivation perspective suggests that violent, aggressive and other forms of negative behavior by detained populations is a direct result of characteristics of the prison environment (Clemmer, 1940; Goffman, 1961; Sykes, 1958). The pains of mass imprisonment – or the deprivation of liberty, autonomy, desirable goods and services, heterosexual relationships, and security (Sykes, 1958) – can result in individuals poorly adapting to the conditions by behaving in negative ways. Studies discuss the conditions of confinement such as, security-level, overcrowding, special programs and violence rate (Daniel & Fleming, 2006; Dye, 2010; Huey & Mcnulty, 2005; Kovasznay et al., 2004; Medlicott, 2017; Mumola, 2005; Way et al., 2005) generally finding support for these environmental factors impacting prison suicide. Suicide occurs most often in maximum-level security facilities, which are more restrictive than their medium and minimum counterparts (Daniel & Fleming, 2006; Huey & McNulty, 2005; Salive et al., 1989; Way et al., 2005). Overcrowding conditions are a direct result of the availability of space as a resource within a facility. Analysis that explores the impact of overcrowding on prison suicide produces mixed findings (Cox et al., 1984; Huey & Mcnulty, 2005). Overcrowding may offer incarcerated people less opportunity to commit suicide as there is an increase in supervision by peers (Huey & McNulty, 2005) or further add to the stress of the carceral environment. Cox et al. (1984) find that facilities ordered by courts to address overcrowding issues were less likely to have experienced suicide than institutions that were not under court-order. Special programs, including those related to rehabilitation, religion, and life skills, may serve as protective activities to prison suicide (Huey & McNulty, 2005; Liebling, 1992; Medlicott, 2017). Lastly, the general level of violence within a facility may correlate with the number of suicides that happen. In other words, violence against others may be an indicator of individuals being more willing to commit harmful acts against themselves. Facilities who experience high levels of violence are subject to cultures that are high in stress and fear (Liebling, 1992). Scholarship also justifies the inclusion of environmental variables when exploring prison suicide.

To date, scholarship exploring prison suicide focuses on characteristics related to incarcerated people and the prison environment. Notably, despite correctional officers’ role in correctional environments, factors related to correctional officers are largely missing in the literature. This is surprising as scholars and practitioners consider correctional officers key in suicide prevention; correctional officer training is the primary way to improve the capability of guardianship (which is often questioned when prison suicide is attempted and/or successful) and reduce suicide (Forrester & Slade, 2014; Slade & Forrester, 2015). In fact, the Behavioral Health Resource Guide provided eight suicide prevention techniques that apply to the correctional setting: staff training, identification/screening, communication, housing, levels of supervision intervention, reporting, follow-up and mortality review (CCHA, 2017). Noteworthy, correctional officers are either directly or indirectly involved in all eight techniques. Additionally, Cramer et al. (2017) concluded from their review and analysis of available literature that training security staff on empirically supported suicide prevention and intervention models and best practices is critical. The centralized focus on correctional staff in these recommended techniques further highlights the crucial role that officers play in the prison experience especially related to prison suicide. Going further, the gendered nature of corrections influences how correctional officers experience and approach work, including how employees interact with incarcerated people at risk of committing suicide. The following section discusses how the gendered nature of corrections and correctional employment is expected to impact prison suicide rates.

### Gender and correctional officers

This study examines the relationship between prison suicide rate and the gender of officers at correctional facilities. It is important to note that although this study uses a gendered approach to conduct a preliminary examination of the role that correctional officer gender has on prison suicide, we acknowledge the fluid, nuanced, and complex nature of gender at the micro-, meso-, and macro-levels. In short, gender is more than a binary variable, rigid, and clear-cut in nature; individuals and institutions may not fit neatly in the concepts presented in this study. Female correctional professionals face unique challenges on-the-job in comparison to their male colleagues. In fact, Walter and Lagace (1999) find female correctional officers are more likely to be unmarried, better educated, younger, less experienced, less interested in the security aspect of corrections, and more satisfied with their work than their male counterparts. These gender-based differences can manifest in employees having varying experiences and assignments that can impact institutional outcomes. This section discusses previous literature on the role of gender in corrections employment. It first reviews gender differences in the execution of work before discussing correctional officer orientations and perceptions.

Gender differences align with the established idea that correctional facilities are masculine institutions that favor traditionally masculine traits over feminine ones (Acker, 1992; Britton, 2006; Kakuk, 2020). In fact, corrections employees often consider their workplace to be a “good old boy” network in which informal relationships between males, particularly White males, have major implications for institutional outcomes (Britton, 1997). These informal connections build social capital that can impact career advancement. In short, promotions for female correctional officers may be more challenging because they often do not have access to the same informal relationships with men, nor the same opportunities to demonstrate correctional leadership/ability (e.g., serving on the Correctional Emergency Response Team (CERT) team) (Britton, 2006). Kakuk (2020) finds that females who work on tactical response teams in Canada report more positive experiences on-the-job when they are assigned to all-female units, as opposed to those assigned to mixed-gender teams. The authors argue that this difference is grounded in the masculine nature of correctional employment, which can restrain female correctional officers to traditional feminine roles (Kakuk, 2020). Britton (1997) also establishes that correctional officer training and assignments assume the officer is male, perpetuating gendered inequities across the institution.

As corrections has increasingly begun to integrate more restorative measures, rather than punitive approaches, correctional training has also shifted to include building skills that equip officers with tools to respond to situations without immediately resorting to physical tactics. In training, for example, correctional recruits are encouraged to master interpersonal communication skills (Burton et al., 2018; Jennings, 1990; Kois et al., 2020). Interpersonal communication skills within corrections can be broken down into three main sections: basic, add-ons, and applications (National Institute of Corrections, 2004). Basic skills include sizing up the situation, observing and listening. Add-ons include ways to respond to detainees by identifying meaning, feeling and content. Lastly, some ways officers can apply interpersonal communication (IPC) skills include handling detainee requests, managing and reinforcing behavior (National Institute of Corrections, 2004). Although all correctional officers must possess skills to engage and respond to detainees when challenges arise, gender may impact how officers apply these skills. Research suggests that female correctional officers are more likely to rely on interpersonal communication skills in stressful times than their male counterparts (Dial et al., 2010; Hogan et al., 2004). Ricciardelli and McKendy (2020) find female correctional officers are more likely to execute their duties by engaging in traits that are culturally considered feminine. The authors identify feminine identity in corrections as both a liability and currency in prison work that fluctuates in meaning depending on the context (Ricciardelli & McKendy, 2020). In all, these studies suggest that male and female officers may rely on skills, such as interpersonal communication and/or traits culturally considered feminine, differently as they execute their jobs. Interacting with incarcerated people who face mental health issues may require approaches that are traditionally and/or culturally considered feminine, thereby making the presence of female correctional officers a form of currency in that circumstance. Because the occurrence of suicide in confinement is increasing and involves a range of critical factors including stress and mental health, we argue that the gendered nature of corrections will hold true for officer impact on prison suicide.

Going further, the gendered nature of corrections may also lead to differences in correctional officer orientations (Gordon, 2006; Hsieh & Boateng, 2020; Stohr et al., 1997). Orientations refer to an individual’s position on something or someone. Scholarship on correctional officers often examines employee orientations on corrections institutions, the profession, and/or the incarcerated population they supervise—in part because orientation of practitioners influences how they perceive and respond to issues and others, resulting in frontline practitioners such as correctional officers acting as ‘street-level bureaucrats’ (SLBs), informally translating policy into practice (Lipsky, 1980). Officers who have a control ideology, ‘law/rule enforcers,’ believe that their primary role is to maintain order to control detainees (Gilbert & Levinson, 1957; Lutze, 2014; Maynard-Moody & Musheno, 2003). In contrast, an officer with a humanistic or ‘social worker’ orientation views incarcerated people with a lens of rehabilitation and optimism who interact with them (Gordon, 2006; Lutze, 2014; Maynard-Moody & Musheno, 2003). SLBs, including correctional officers and support staff, tend to gravitate to one orientation over the other, but most experience role conflict and adaptions, which are influenced by individual- and organizational-level conditions, including available cultural or discretionary toolkits (Rizzo et al., 1970; Swidler, 1986; Taxman & Belenko, 2012 Whittle, 2018). The orientation of correctional officers – whether controlling rule enforcer or humanistic social worker – directly relates to how they perform their job and the experiences of incarcerated people. In short, having more humanistic-oriented officers on staff may improve officer interactions with detainees, including those who have mental health issues. Officer orientations may be gendered as well. A study exploring the control ideology of correctional officers found that African Americans and female officers were more humanistic than their White and male counterparts, respectively (Gordon, 2006). This research suggests that how correctional facilities maintain order and interact with detainees is related to officer gender. More recently, White et al. (2015) studied probation officers finding female employees to be more competent with mental health issues than males. Additionally, officers who viewed their work as more treatment-oriented opposed to enforcement-oriented experience greater burnout. These studies support that increasing the presence of female and African American officers may lead to a more rehabilitative workforce, because both of these groups are more likely to optimistically interact with the incarcerated population.

The professionalization of correctional employees also includes security and service orientations (Stohr et al., 1997). The security orientation focuses on ensuring safety within a facility through order and control. In contrast, the service orientation emphasizes the importance of meeting the needs of incarcerated people both during confinement and in preparation for reintegration into society. Related to suicide in confinement, officers invested in the service orientation may approach incarcerated persons at risk of harming themselves differently in times of crises than their more security-oriented counterparts. Some studies find that female correctional officers are more likely to align with the service orientation rather than security (Feingold, 1994; Jurik, 1985; Jurik & Halemba, 1984; Walters, 1992). However, other empirical literature finds no major differences in orientation styles across genders (Stohr et al., 1997), and more recently, contrary to prior research findings on officer orientations, Rudes et al. (2017) find that female officers regardless of age or rank are more likely than male officers to employ confrontational strategies with inmates.

Like the literature on the impact of gender on the orientations of correctional officers, scholarship on perceptions of correctional officers also finds mixed support for the significance of gender. In fact, Stohr et al. (1997) suggest that being employed in corrections serves as a master status, muting any potential gender differences in perceptions. Scholarship backs this claim. Hsieh and Boateng found no significant gender differences in how Ghanaian correctional officers perceive distributive justice (fairness in the allocation of resources) and procedural justice (fairness in processes) within facilities (2020). Correctional officer perceptions of distributive and procedural justice relate to how they interact with incarcerated people, including those who may be at risk of attempting self-harm, because they relate to how the institution responds to issues related to equity, equality, needs (distributive), policies, procedures, and processes (procedural). In other words, correctional officers who believe the organization is unjust may respond differently to incarcerated individuals, particularly in times of crisis. Other studies that demonstrate the impact of gender on correctional officer perceptions are a bit more nuanced. Hemmens et al. (2002) examined how correctional officers of different genders perceive female correctional officers, finding that while most employees share similar views on all issues, regardless of gender, male correctional officers with former military experience are more likely to hold negative perceptions of female correctional officers. Additionally, female correctional officers are more likely to hold positive perceptions of other female correctional officers (Hemmens et al., 2002). Therefore, while the perceptions of corrections professionals are similar, studies demonstrate that there are pockets of difference based on gender.

In all, previous scholarship gives mixed support for the impact of officer gender versus organizational dynamics in corrections work. While organizations shape the orientation and perceptions of officers, gender matters when examining the way individuals perform work, relying on different skills. Beyond gender, there is also empirical evidence that the racial makeup of correctional officers may influence approaches to work, including orientations (Britton, 2006; Gordon, 2006). Although gender is the focus of this study, the impact of these intersecting identities in the literature (Gordon, 2006) justifies additional empirical analysis that considers how both correctional officer gender and race impact prison suicide.

## Contribution

This study provides a preliminary examination of the relationship between prison suicide and correctional officer gender. Previous studies on prison suicide focus on importation and deprivation factors related to incarcerated people. However, prior literature overlooks the impact that correctional officers have on the prison environment, especially the role of officer gender. This is surprising as literature establishes the masculine nature of corrections, and the influence correctional officer gender has on work. Scholarship suggests that female officers may be more likely to rely on traditionally feminine traits to navigate their jobs (Ricciardelli & McKendy, 2020), hold more humanistic/service orientations (Gordon, 2006), and perceive other female officers as capable (Hemmens et al., 2002), compared to their male counterparts. This study seeks to understand the impact of correctional officer gender on prison by addressing the research question: What is the relationship between prison suicide rates and correctional officer gender?

## Data & methodology

This study uses data from the 2000 Census of State and Federal Adult Correctional Facilities (CCF). This national level data set occurs every 5–10 years by the Bureau of Justice Statistics (BJS), providing detailed information on the types of detainees housed, characteristics of the prison facility, and staff demographics. Noteworthy, questionnaires change during each distribution of the CCF, resulting in differences in the data collected. Although the most recent CCF occurred in 2019, the 2000 CCF is the latest rendition to incorporate items about death in custody, prison suicide, staff suicide training, and overall staff characteristics. Therefore, although two decades old, this dataset is the most appropriate for the exploration of the research question.

The 2000 CCF was the 6th rendition of the project and included 1,668 facilities; the unit of analysis is facility. BJS distributed the surveys via mail; the ultimate response rate was 100%. Items on the questionnaire referred to a one-year period, between July 1st, 1999, and June 30th, 2000. Respondents could skip some questions on the survey and estimate values if the exact number was unknown. Survey items with the best response rates were those identified as “critical data items” and included the physical security level of the facility as well as the percentage of detainees permitted to leave the facility. Due to lack of response on key variables of interest, the analysis uses listwise deletion to handle missing data. The total number of facilities in our analysis was 916; however, some facilities failed to answer all questions, particularly those related to demographics of correctional officers. Despite its imperfections, this dataset is appropriate for the examination of prison suicide. Furthermore, the data are rare for three reasons: (1) it is a national survey of corrections facilities; (2) it includes items related to both correctional employees and incarcerated persons; and (3) it includes detainee suicide data.

## Measures

### Dependent variable

The questionnaire asked for the number of suicides by gender of incarcerated persons that occurred within each facility over a one-year period. These gender-based items produced a single, count variable. The dependent variable is the number of suicides that a facility experienced within one year.

### Independent variables

#### Importation: incarcerated individuals

Considering previous literature, this study includes the following detainee/individual-level variables: gender composition, whether the facility housed juvenile(s) (yes = 1), percentage of White detainees, and percentage of incarcerated persons receiving mental health treatment.^2^

#### Deprivation: incarcerated individuals

This study includes five environmental variables used in previous literature: security-level, the ability to leave facility grounds, overcrowding, facilities under court order for overcrowding, and violence rate.

Security-level is measured on a 4-point scale, with 3 being super maximum security and 0 being minimum security. Akin to security-level, some institutions in the sample allowed detainees to leave campus for a certain amount of time. The ability to experience conditions beyond confinement may also relate to prison suicide and is considered in this study using a dichotomous variable (Yes = 1). Overcrowding is calculated by dividing the number of detainees by the rated facility capacity then multiplying by 100. Rated facility capacity is the number of beds the facility is permitted to have according to a correctional official. We use rated capacity rather than design capacity to account for changes in corrections (incarcerated population size, policies/procedures related to housing, etc.). Lastly, the violence rate is the number of assaults on staff and detainees committed by incarcerated people divided by the incarcerated population on June 30^th^, 2000.

#### Correctional officers

Two variables capture how correctional officer characteristics may influence prison suicide that focus on correctional officer gender and race. Demographics of correctional officers were reported as counts and calculated as percentages using the total count.

Two variables measure the prison environment as it related to correctional officers may impact prison suicide: ratio of detainees to correctional officers and training directly related to suicide prevention. First, understaffed and/or overcrowded prisons can increase stress and impact the way officers do their jobs. Second, the survey instrument also inquired about the number of suicide procedures/trainings they had available for correctional officers including assessment of risk at intake, staff training in risk assessment/suicide prevention, special counseling, or psychiatric services, live or remote monitoring of high-risk detainees, suicide watch cell or special location and suicide prevention teams. Both factors may impact officer interactions with incarcerated persons who are at risk of committing suicide.

#### Control variables

This study includes three control variables that directly related to the organization’s environment that may impact prison suicide: source of funding, age of facility and number of incarcerated people housed in the facility on June 30, 2000.

Whether the state or a private backer supported correctional facility captures broad structural differences in resources, administration, and other possible institutional variation because of funding sources. Additionally, differences in architecture and the health of the physical structure of the institution may influence behavior and management decisions; therefore, prison age is considered. Lastly, the number of incarcerated persons housed within each institution is also directly related to the number of suicides that a facility may experience as an increase in detainees increases the risk of the outcome. Therefore, the analysis also controls for the number of detainees as of June 30^th^, 2000.

## Analytic strategy

This study uses a series of analyses to explore the relationship between prison suicide rates and correctional officer gender. Descriptive statistics outline the distribution of the variables of interest, providing an overview of the data. A series of negative binomial regression models demonstrate how factors related to individual traits (importation theory), organizational characteristics (deprivation theory), and correctional officer demographics (focusing on gender) impact prison suicide rates. Finally, negative binomial models including interaction terms were run considering prior literature finds correctional officer gender and race to be influential in their orientation (Gordon, 2006). Because prison suicide is a rare occurrence, and the data are overdispersed, it is appropriate to use negative binomial regression models (Hilbe, 2011). Additionally, to compare the prison suicide count across facilities, the number of incarcerated people serves as an exposure variable in the negative binomial models (Hilbe, 2011). Including the exposure variable allows the number of suicides to be compared across facilities as if it were a rate because it takes the number of incarcerated people into account.

## Results

### Descriptive statistics

Table 1 lists descriptive statistics for each variable included in analysis. Of the 916 correctional institutions included in our analysis, 8% were private facilities. Facilities ranged from 18–207 years old, with an average of 50 years. Eighty-three percent of facilities reported no suicides from June 1999- June 2000. Only three facilities experienced four suicides over one year, the highest value reported.

**Table 1:** Descriptive Statistics

	**N**	**Mean**	**Std. Deviation**	**Minimum**	**Maximum**
**Dependent Variable**					
Number of Suicides	915	.1672	.4988	0	4
**Independent Variables**					
**Importation**					
All Female Institution	915	.0896	.2858	0	1
Juveniles? (Y = 1)	915	.3421	.4747	0	1
% White Detainees	915	39.1939	18.3158	0	97.3684
% Detainees receiving Mental Health Services	915	41.2396	38.4752	0	100
**Deprivation**					
Percent of Detainees who Leave Facility	915	.8284	.3772	0	1
Security-level	915	1.0514	.7818	0	3
Overcrowded? (Y = 1)	915	.2940	.4558	0	1
Court-ordered Overcrowded? (Y = 1)	915	.1016	.3023	0	1
Violence Rate	915	2.7639	4.6487	0	56.8182
**Correctional Officer Related**					
Percent White Officers	898	67.4884	28.5746	0	100
Percent Female Officers	898	25.0294	17.1060	0	92.7711
CO: Incarcerated Person Ratio	915	23.9502	10.9442	0	81.4685
Number of Officer Suicide Prevention Programs	915	4.1683	1.5676	0	6
**Control**					
Private? (Y = 1)	915	.0808	.2728	0	1
Age of Prison (Years)	915	50	33	18	207
Number of Detainees	915	1089	1010	11	7223

Eighty-four percent of institutions housed all males, followed by nine percent all females and seven percent mixed gender. On average, 39% of the detainee population were White at a given facility. Thirty four percent of facilities housed juveniles, individuals under 18 years old. On average, 41% of the incarcerated population were receiving mental health treatment. Twenty nine percent of facilities housed more incarcerated people than recommended by a rating official, deeming them overcrowded. And of all respondents, ten percent received court orders to address issues of overcrowding, signaling a major problem.

On average, institutions employed correctional officers that were 68% White. Institutions reported a quarter (25%) of correctional officers as female on average. Data on intersecting identities of officers (e.g., Black women, White men) were unavailable which limits the way intersecting identities can be examined in this study. Institutions averaged between 4–5 suicide prevention procedures/programs, three percent reported zero programs while 16% reported six (the maximum).

### Negative binomial results

Table 2 shows the results of a series of negative binomial models which predict the expected likelihood of changes in the suicide rate at an institution. Negative binomial regression is the appropriate model because the count includes mostly zeros and is overdispersed. Again, the number of detainees in each facility is used as an exposure variable, as an increase in the incarcerated population leads to a greater risk of prison suicide.

**Table 2:** Negative Binomial Regression: Number of Prison Suicides

	Model 1: *N* = 915*		Model 2: *N* = 915*		Model 3: *N* = 898*	
	IRR	Std. Error	IRR	Std. Error	IRR	Std. Error
**Importation**						
All Female Institution	.73	.32	.70	.32	1.12	.54
Juveniles? (Y = 1)	1.07	.20	.93	.20	1.02	.19
% White Detainees	1.00	.01	1.00	.01	1.01	.01
% Detainees receiving Mental Health Services	1.00	.00	1.00	.00	1.00	.00
**Deprivation**						
Detainees Leave Facility	-	-	1.32	.24	1.42	.22
Security Level	-	-	2.13*	.31	1.98*	.29
Overcrowded? (Y = 1)	-	-	1.05	.30	1.10	.32
Court-ordered Overcrowded? (Y = 1)	-	-	1.46	.45	1.87	.60
Violence Rate	-	-	1.04*	.01	1.04*	.01
**Correctional Officer Related**						
Percent White Officers	-	-	-	-	.99	.00
Percent Female Officers	-	-	-	-	.98*	.01
CO: Inmate Ratio	-	-	-	-	1.02*	.00
Number of Officer Suicide Prevention Programs	-	-	-	-	1.03	.08
**Control**						
Private? (Y = 1)	.39	.23	.59	.40	.82	.53
Age of Prison (Years)	1.01*	.00	1.01*	.00	1.00*	.00
Number of Detainees	*(Exposure)*		*(Exposure)*		*(Exposure)*	
	*Pseudo R2:.0212*		*Pseudo R2:.0787*		*Pseudo R2: 0894*	
	*LR chi2(6):16.35*		*LR chi2(11): 60.62*		*LR chi2(18): 68.54*	
	*Log Likelihood: -376.8725*		*Log Likelihood: -354.7373*		*Log Likelihood: -349.2086*	

The asteriks "*" symbolize statistical significance where p≤.05

Model 1 includes importation variables related to incarcerated persons. In this model, only the control variable, age of prison, is statistically significant, finding that expected suicide rates will slightly increase as facilities age.

Model 2 includes importation and deprivation variables related to the incarcerated population. Even when other organizational controls are added to the model, importation variables remain statistically insignificant; prison age remains influential in the positive direction. Institutions with more security are expected to see an increase in suicide rate by 113%. As the violence rate of an institution increases by 1% it is expected that the suicide rate will increase by 4.3%.

Model 3 introduces correctional officer-related variables into the regression. Organizational characteristics such as prison age, security-level, and violence rate continue to have statistically significant positive relationships with the expected number of prison suicides. Facilities ordered to address overcrowding by courts, were expected to have a suicide rate 87% higher than those who were not.

Organizational variables related to correctional officers’ work environment; the analysis reveals that as the ratio of detainee-to-officer increases the expected suicide rate also increases by 1.7%. The percentage of White correctional officers was not statistically significant. This means that the racial demographic makeup of correctional officers did not have a significant impact on the expected prison suicide rate. Directly addressing the research question, Table 2: Model 3 supports the hypothesis of a negative relationship between correctional officer gender composition and prison suicide. As the percentage of female officers increases, the expected suicide rate within institutions decreases by 2%. This finding supports the idea that the presence of female correctional officers impact prison suicide.

Table 3 shows two supplemental negative binomial regression models that include interaction terms. Model 4 includes an interaction term for correctional officer race and gender. This is grounded in scholarship that suggests Black and female correctional officers will have a more humanistic approach to work than their White and male counterparts, respectively (Gordon, 2006). Although the model was statistically significant and the reported findings from model 3 remained the same, the interaction term was not. Once again, variables related to the organization such as security level, court order for overcrowding, violent incidents, and prison age had statistically significant positive relationships with prison suicide rate. Additionally, as the detainee-to-officer ratio increases the expected suicide rate also increases. And as the percentage of female officers increases, the expected suicide rate decreases by 3%.

**Table 3:** Negative Binomial Regression: Number of Prison Suicides – Supplemental Model

	Supplemental Model 4: *N* = 898*		Model 5: *N*= 898*	
	IRR	Std. Error	IRR	Std. Error
**Importation**				
All Female Institution	1.1053	.58	.0655	.10
Juveniles? (Y = 1)	.9809	.18	1.0346	.19
% White Detainees	1.0064	.01	1.0084	.01
% Detainees receiving Mental Health Services	1.0014	.00	1.0018	.00
**Deprivation**				
Detainees Leave Facility	1.3819	.44	1.3980	.44
Security Level	2.0377*	.30	1.9894*	.29
Overcrowded? (Y = 1)	1.0447	.21	1.0806	.21
Court-ordered Overcrowded? (Y = 1)	1.9589*	.51	1.9908*	.51
Violence Rate	1.0393*	.02	1.0367*	.02
**Correctional Officer Related**				
Percent Black Officers	.9994	.01	-	-
Percent White Officers	-	-	.9915*	.00
Percent Female Officers	.9735*	.01	.9734*	.01
CO: Inmate Ratio	1.0164*	.01	1.0189*	.01
Number of Officer Suicide Prevention Programs	1.0151	.08	1.0245	.08
Percent Black CO * Percent Female CO	1.0002	.00	-	-
All Female Institution * Percent Female CO	-	-	1.0544*	.03
**Control**				
Private? (Y = 1)	.8255	.51	.8899	.55
Age of Prison (Years)	1.0041*	.00	1.0044*	.00
Number of Detainees	*(Exposure)*		*(Exposure)*	
	*Pseudo R2:.0886*		*Pseudo R2:.0960*	
	*LR chi2(16): 67.92*		*LR chi2(16):73.66*	
	*Log Likelihood: -349.5199*		*Log Likelihood: -346.6487*	

The asteriks "*" symbolize statistical significance where p≤.05

Model 5 includes an interaction term for the gender of incarcerated people housed in the facility and the gender of correctional officers. Results aligned with previous findings in terms of security level, court order for overcrowding, violent incident, detainee-to-officer ratio, prison age, each having a statistically significant positive relationship with the prison suicide rate. Again, these findings reflect the power that the environment of corrections organizations has on prison suicide. Additionally, as the percentage of female officers increased, the expected suicide rate drops by 3%. The interaction term is statistically significant, signaling that as the percentage of female officers increases within all female facilities the expected suicide rate increases by 5.5%. That is, an increased presence of female officers is expected to increase the suicide rate in facilities that house all women.

**Fig. 1:**
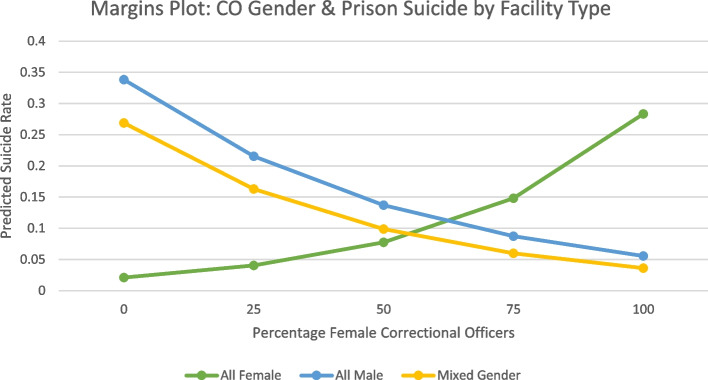
This graph depicts a margins plot examining correctional officer binary gender and prison sucidie occcurences by facility type

Considering the statistical significance of the interaction term in Model 5, we conduct a series of margins plots to further examine the relationship. Margins plots are a visual depiction of an expected outcome under specific conditions. In this case, margins plots provide a graphical representation of the expected suicide rate in all-female, all male, and mixed gender facilities when the percentage of female officers equals zero, twenty-five, fifty, and one hundred percent. Results demonstrate that for all-male and mixed gender facilities, as the percentage of female correctional officers increases the expected rate of suicide decreases. However, in all female prisons, as the percentage of female correctional officers increases, the expected rate of suicide also increases (Fig. 1).

Placing this finding within the context of corrections, legislation involving detaining incarcerated women make the presence of female correctional officers mandatory to complete certain tasks such as strip searches (Smith and Loomis, 2013). Considering this, our findings suggest that gender diversity of correctional officers matters across contexts, rather than just increasing the presence of female officers.

## Discussion

Our results find that gender diversity among officers is associated with a reduction in the rate of prison suicide. Previous scholarship primarily focused on how correctional officer training and individual- and environmental-factors influenced prison suicide. This work fills a gap in the literature by acknowledging the complex role correctional officers play in the prison environment beyond training. That is, this study is one of the first to explore how who correctional officers are – rather than solely what they do – impacts prison outcomes, specifically prison suicide. Results show that variables related to deprivation and correctional officer demographics significantly influence prison suicide. Importation factors were not significantly related to prison suicide. Directly addressing the research question, having a mixed gender correctional officer workforce is critical in reducing the occurrences of prison suicide.

Additionally, the likelihood of suicide decreases as the ratio of correctional officers to incarcerated people levels out, resulting in an increase of supervision. It is noteworthy that the number of suicide prevention programs that an institution has in place did not significantly predict the likelihood of suicide in the multivariate model. The influence of correctional officer demographics rather than the extent of training programs may be surprising, especially since research and professional organizations often suggest an increase in suicide prevention curricula to address detainee self-harm. This may be because training programs are instituted as a response to high suicide rates, rather than as a purely preventative measure. Additionally, training programs for correctional officer response to inmate suicide may be ineffective and/or a relatively small part of the overall preparedness courses they receive. Future research is encouraged to evaluate these programs for effectiveness and other factors.

This study also finds organizational-level deprivation variables are consistently important in understanding prison suicide and should shape prevention strategies. Overall, higher-level security facilities are more likely to experience prison suicide than their counterparts. Similarly, institutions with higher rates of violence and have been court ordered to address overcrowding are more likely to experience prison suicide. These results align with previous studies which find a positive and statistically significant correlation between detainee-related conflicts and prison suicide (Kovasznay et al., 2004; White et al., 2002). Although literature supports the deprivation perspective, it is important to note that conditions of confinement vary across institutions (Haycock, 1993). In short, the incarceration experience varies and does not solely account for a detainees’ decision to commit suicide. However, this study adds to existing scholarly justification to continue including deprivation factors in future work.

While this work contributes to the literature by providing insight on correctional officers and prison suicide, there are limitations. As mentioned above, the CCF data is solely at the institution level, using aggregates of individual factors to inform their influence on suicide. Studies demonstrate the importance of individual-level variables as well as the perspective of the detainee, on prison suicide (Daniel & Fleming, 2006; Way et al., 2005). Although a few studies exist that include both micro- and macro- level data (Dye, 2010), this study suggests future research collect individual, environment, and correctional officer data to show a fuller picture of the prison environment. Another variable for future studies to consider is the overall level of education of correctional employees rather than solely training on suicide, as this may impact how staff interact with incarcerated people, including those at risk of engaging in suicidal behavior. Additionally, the institutions surveyed were adult facilities, although 68% housed at least one juvenile. Scholarship shows that younger individuals are more likely to commit suicide than older persons (Daniel & Fleming, 2006). Therefore, analyzing juvenile facilities may provide noteworthy distinctions from and/or confirm results found in adult prisons. Another limitation is that the section on staff and detainee health on the CCF was optional. For example, no federal facilities provided information for these items. This may suggest a sample bias for the dependent variable. Future survey instruments should consider the health of prisoners and correctional employees a priority by making the items mandatory for institutions to complete. Although missing data will continue to be an issue, the instrument should highlight the importance of health within the prison environment. It is also important to acknowledge the fluid nature of gender. Rather than being a binary variable, gender is a complex concept. The nature of this dataset reduces gender to a measure of biological sex. Future research should explore the role of correctional officer gender on prison outcomes via qualitative methods to provide more nuance. Additionally, rather than being a definitive finding on the relationship between CO gender composition and prison suicide rates, this study points to the need for further research on prison organization, gender dynamics and suicide among incarcerated people. Lastly, the CCF data are from 2000. The 2000 CCF is the last to collect data on both prison suicide *and* officer demographics for correctional institutions nationwide. Although aged, these data provide rare and critical insight into a growing and alarming health problem. In early 2019, the Bureau of Justice Statistics accepted comments on the importance of the CCF in practice, research, and policy. BJS also welcomed suggestions regarding improving data collection efforts. The lead author submitted a comment highlighting this research, key findings and the importance of reintegrating suicide, training, and other health-related outcomes in future CCF data collection efforts.

### Implications

Overall, the findings from this study support a range of translational implications for scholarship and practice. First, researchers and practitioners are encouraged to view correctional officers as being able to both contribute to and help resolve challenges that occur in the carceral environment. Current correctional approaches to suicide prevention are based on studies and data that prioritize the experiences of individuals, structural characteristics of prisons, and officer training programs (CCHA, 2017; Danto, 1997). Scholars and practitioners should evaluate current suicide prevention programs to determine their effectiveness as results from this study find the number of programs statistically insignificant in impacting suicide rates. It is particularly important to assess whether programs are more reactive than preventative.

Analyses from this study provides insight that gender diversity among correctional officers may reduce prison suicide rates. That is, these results suggest new pathways for further research on suicide prevention. Future research should consider how the demographic makeup of correctional officers and staff (social work, counselors, educators, etc.) has on incarcerated outcomes including those related to health, recidivism, violence, and the coping strategies that detainees adopt to adjust to incarceration. Further, when possible, a mixed methodological approach is recommended to allow for nuances that quantitative data does not allow.

This research suggests that human resources departments – especially the recruitment, support, and retention of diverse employees—may be key in shaping overall institutional outcomes. By acknowledging the importance of officer gender on the prison environment, this study provides more empirical support for the value of women in corrections. Corrections institutions should be mindful that the effect of gender in the work environment persists beyond training and other types of officer socialization. Finally, despite the lack of statistically significant effects for staff racial combination in my analysis, other research (Gordon, 2006) suggests the importance of racial diversity among prison staff. Future research should explore a range of correctional officer demongraphics on carceral outcomes.

## Conclusion

Although women have worked in corrections for nearly 150 years, their ability and belonging in the prison environment is debated. This study finds that gender diversity in correctional settings can influence organizational outcomes. Specifically, officer gender diversity at all-male, mixed-gender, and all-female facilities leads to a decrease in prison suicide rates. Future research is encouraged to explore how correctional officer demographic composition influences other prison outcomes. Therefore, theoretical frameworks used to understand correctional outcomes must be inclusive of employee presence, demographics, and other related variables to explore corrections in a comprehensive way. For practitioners, the focus on recruiting, supporting, and retaining diverse correctional officers is key to promote positive outcomes. In sum, this work emphasizes that correctional officers are not a monolithic group and that considering the role of their identity on shaping the prison environment may lead to improved outcomes. The presence of both male and female officers in a facility impacts prison suicide.

## Data Availability

The 2000 Census of State and Federal Adult Correctional Facilities, 2000 Data analyzed in this study are publicly available at https://www.icpsr.umich.edu/web/NACJD/studies/4021.

## Footnotes

1 This paper examines the importance of organizational versus individual characteristics in explaining inmate suicide rates and the degree to which officer characteristics (i.e., gender) might impact the prison environment and thereby reduce suicide rates. It is critical to note that when discussing suicide within the incarcerated population, literature to date has relied on the terms “inmate suicide” and “prison suicide”. In the ladder portion of this study, we use the term inmate suicide as that is the term used in existing scholarship and the term used in the data collection instrument; however, we also acknowledge the shift in language to reflect the humanity of incarcerated people beyond their status as detained.

2 The item that captured the number of incarcerated persons receiving mental health treatment did not provide details on these services; it was captured yes/no/not applicable/don’t know or missing.
